# Acute infarct of the corpus callosum presenting as alien hand syndrome: evidence of diffusion weighted imaging and magnetic resonance angiography

**DOI:** 10.1186/1471-2377-11-142

**Published:** 2011-11-09

**Authors:** Jun Liang Yuan, Shuang Kun Wang, Xiao Juan Guo, Wen Li Hu

**Affiliations:** 1 Department of Neurology, Beijing Chaoyang Hospital, Capital Medical University, No. 8 South Gongti Road, Beijing 100020, China; 2 Department of Radiology, Beijing Chaoyang Hospital, Capital Medical University, No. 8 South Gongti Road, Beijing 100020, China

## Abstract

**Background:**

Infarcts of the corpus callosum are rare and have not been well documented previously. As for a variety of signs and symptoms presented, alien hand syndrome (AHS) can be easily overlooked.

**Case presentation:**

In this report, we present a patient with a mixed types of AHS coexistence secondary to the corpus callosum infarction, including a motor type of AHS by intermanual conflict (callosal type AHS) and a sensory type of AHS by alien hand and left hemianesthesia (posterior AHS).

**Conclusions:**

Our case may contribute to the early recognition of AHS and to explore the abnormal neural mechanism of AHS. To our knowledge, rare reports have ever documented such mixed AHS coexisting secondary to the callosal lesion, based on advanced neuroimaging methods as in our case.

## Background

Alien hand syndrome (AHS) is a rare clinical syndrome, characterized by involuntary, uncontrollable and purposeless movements of one upper limb, and the patient denies ownership of the limb when touching it without visual guidance [1]. Such movements are aimless and differentiable from choreic and athetotic movements [2]. AHS is usually considered as a type of interhemispheric disconnection syndrome resulting from several lesions involving supplementary motor area, corpus callosum, medial frontal cortex, frontal, posterior parietal [3], or thalamus [4]. However, the neural mechanisms of AHS have remained controversial. Here, in this report, we describe a recent case of mixed types of AHS secondary to the infarction of the corpus callosum. Examinations of 3-Tesla magnetic resonance imaging (MRI) and magnetic resonance angiography (MRA) were used to assist early diagnosis of such a callosal infarct and to identify the abnormal neural processes of AHS. We briefly discuss the complex varieties of AHS, their anatomic substrates and the underlying neural mechanisms. To our knowledge, rare reports have documented such mixed types of AHS coexistence secondary to the corpus callosum infarction based on MRI, as reported in our case.

## Case presentation

A 71-year-old right-handed woman was admitted to our hospital because of urgent onset of paraphasia and slight weakness of her left hand for 13 days. The patient had a 20-year history of hypertension, coronary heart disease and insulin-dependent diabetes mellitus, with no history of drinking or smoking, and negative personal or family history of neurological diseases. Educational history of the patient was five years.

She complained of involuntary and purposeless movements of the left hand and abnormal feelings in the left upper limb. She claimed that the left hand did not belong to herself but to her grandson (alien hand). She also presented that her left hand was controlled by someone else instead of herself, and the two hands always "fought" with each other during bimanual coordination, as the left hand performed movements opposite to the right hand (intermanual conflict). For instance, when she used her right hand to unbutton her clothes, the left hand interfered by buttoning the clothes up. Also, she had visual hallucination at admission, such as her grandson standing beside her bed. Although frustrated with her alien hand, she was not afraid of her abnormal hand.

On her bedside mental examination, she had no symptoms of amnesia, disorientation, agraphia, tactile naming in left hand, alexia in left visual field, hemispatial neglect and hemiballismus. The scale of the Mini-mental state examination was 24 when she was admitted to our hospital. She comprehended language well, but could not find appropriate words to answer questions, frequently using wrong words. Left hemiasomatognosia and apraxia were also noted. For instance, she did not perceive the existence of her left hand, with the abnormal perception that it was her grandson's hand (hemiasomatognosia). She had apraxia when miming how to use tools with her left hand, such as dressing, brushing teeth, combing her hair (ideomotor apraxia). She also had some difficulties in purposefully picking up objects (motor apraxia). A neurological examination showed mild left hemiparesis with slight clumsiness, and left hemianesthesia. Babinski sign was positive on the left. Activities of daily living (ADLs) were severely impaired, such as bathing, dressing and transferring, but the self feeding, bladder and bowel control were not significantly affected. The impairment for ADLs may be attributed to the left hemiparesis, apraxia, and intermanual conflict, with their disabling impact on everyday life.

**Figure 1:**
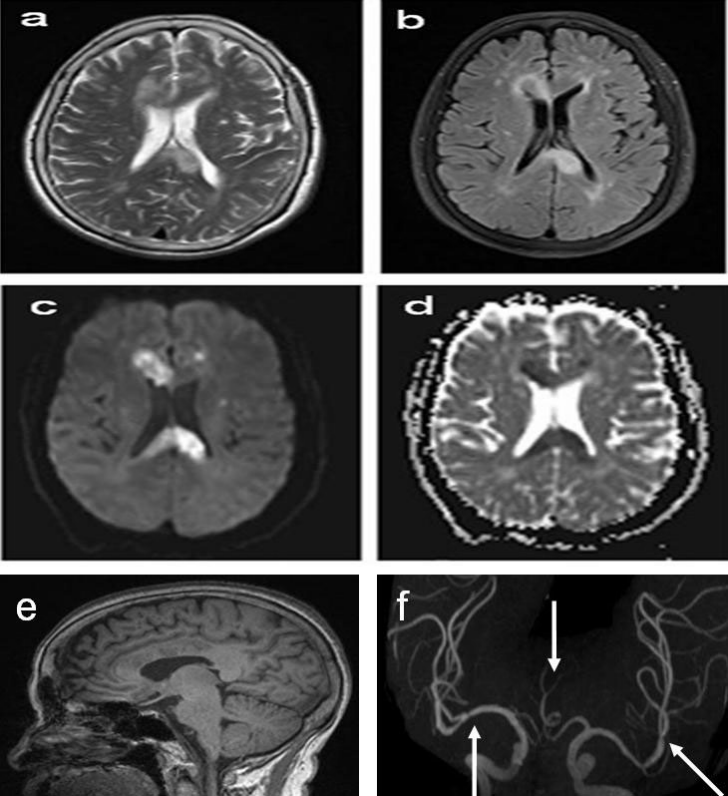
**Multi-modal brain MRI**. Axial T2-weighted (a) and FLAIR images (b) depicted T2 hyper-intensity and sagittal T1-weighted MRI (e) showed hypo-intensity in the genu, body and splenium of the corpus callosum. Axial DWI demonstrated high signal intensity in the observed T2 abnormality of the lesions of the corpus callosum (c). Corresponding ADC map illustrated matched abnormality, confirming restricted diffusion in the corpus callosum (d). MRA showed the absence of the A1 segment of the bilateral anterior cerebral artery. Atherosclerotic segment of stenosis in the area of the bilateral middle cerebral artery and left posterior cerebral artery were noted (arrows) (f).

**Figure 2:**
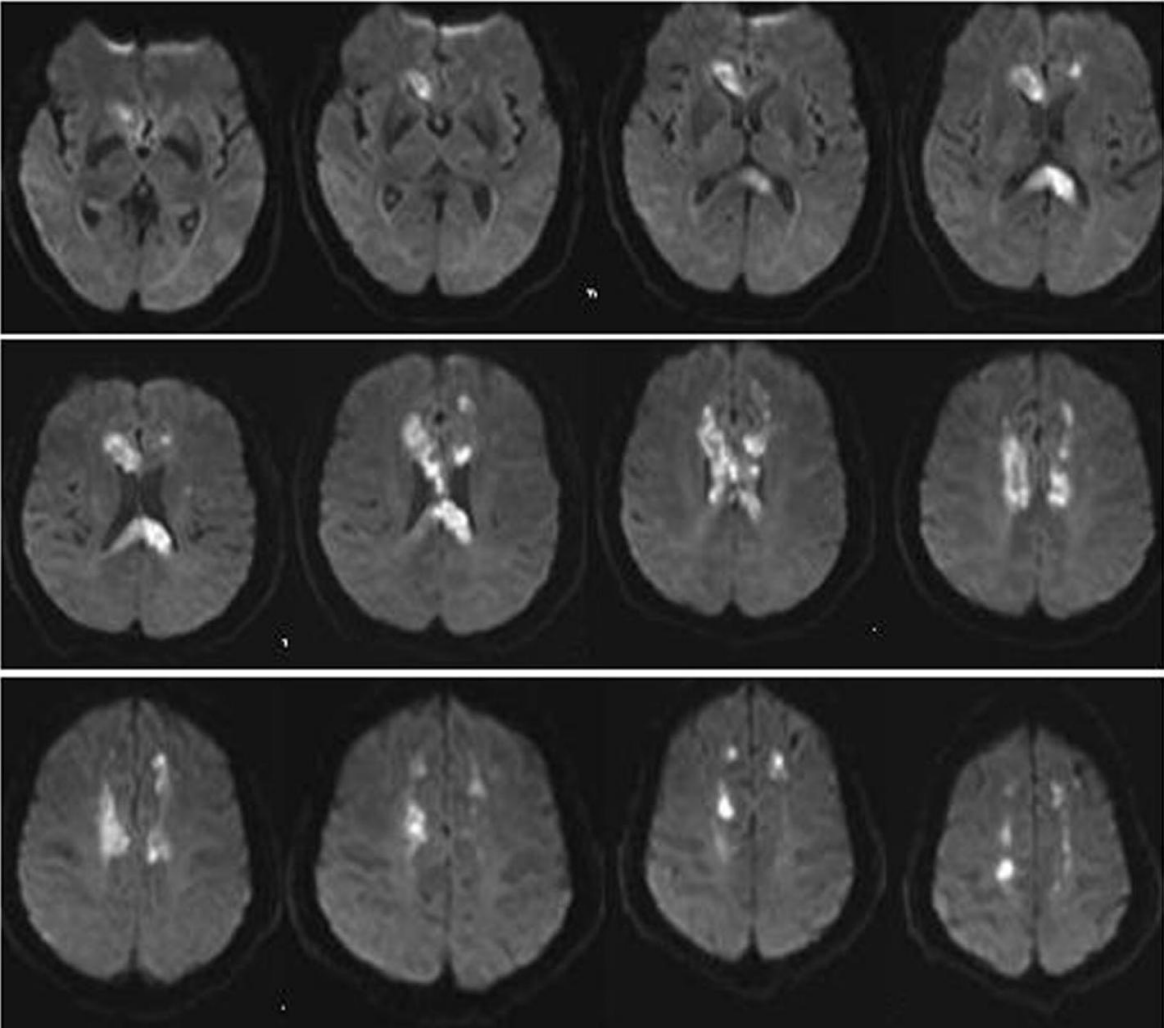
**Axial DWI showed high signal intensity in all the slices of the lesions of the corpus callosum**.

Carotid ultrasound demonstrated atherosclerosis plaques bilaterally in carotid artery. A brain 3-Tesla MRI was performed 3 days after admission, including conventional MR sequences: sagittal T1, axial T2-weighted, axial fast fluid attenuated inversion recovery (FLAIR), along with diffusion weighted imaging (DWI). Conventional sequences of MRI indicated regions of low signal intensity in the genu, body and splenium of the corpus callosum on the T1 weighted image (Figure 1e) and high signal intensity in the same area on T2 weighted and FLAIR images (Figure 1a-b). DWI demonstrated high signal intensity in the observed T2 abnormality of the lesions, with corresponding restricted diffusion on the apparent diffusion coefficient (ADC) maps (Figure 1c-d, Figure 2). MRA showed the absence of the A1 segment of the bilateral anterior cerebral artery (ACA). Atherosclerotic segment of stenosis in the area of the bilateral middle cerebral artery (MCA) and left posterior cerebral artery (PCA) were also noted (Figure 1f).

Two weeks later after treatment (platelet aggregation inhibitor and rehabilitation), the patient's aphasia, apraxia and hemiasomatognosia disappeared and ADLs were significantly improved. At discharge, this patient could perform routine activities independently and the behaviors of AHS were completely resolved.

## Discussion

We described a recent case of an acute infarct of the corpus callosum presented with mixed types of AHS. AHS was originally described by Brion and Jedynac in 1972, characterized by the denial of ownership of one hand when it was held by the other hand behind the back, due to the lesions of the corpus callosum [5]. Two types of AHS were proposed by Feinberg in 1992, including frontal and callosal types [6]. Frontal AHS always occurs in the dominant hand, characterized by reflexive grasping, groping, and compulsive manipulation of tools, and is usually associated with frontal release signs. The supplementary motor area, anterior cingulate gyrus, medial prefrontal cortex and anterior corpus callosum have been described in this type of AHS [7]. The second type or callosal AHS is characterized primarily by the presence of intermanual conflict and the absence of frontal release signs. This type of AHS occurs in the non-dominant limb and may result from only a corpus callosal lesion [8].

Based on previous classifications, Kato et al reported three types of AHS [9]. The first and second types were similar to the frontal and callosal type of AHS, as described by Feinberg. The third type of AHS or posterior AHS was due to lesions in cortical and subcortical structures supplied by the posterior cerebral artery, characterized by sensory impairment such as left hemianesthesia, left homonymous hemianopia and left spatial neglect. The first two types of AHS are classified as an "anterior" or "motor" form of AHS, whereas the posterior AHS is considered as a "sensory" form of AHS. However, Aboitiz et al strongly proposed that the cases of AHS should be classified into at least five broad categories [10]: diagonistic dyspraxia and related syndromes, alien hand, way-ward hand and related syndromes, supernumerary hands, and agonistic dyspraxia.

Although different subtypes of AHS have been distinguished, these classifications obviously can not involve the wide clinical variety of abnormal behaviors of the upper limb. Up to now, the classifications of AHS are still inconsistent and have been remain disputable. It is regrettable because different pathophysiological mechanisms probably underlie the diverse behaviors in alien hand. As for lack of uniformity in assessment methods (behavioral tests, neuroimaging), Scepkowski indicated it may be difficult to establish clear subtypes of alien-hand phenomena [11].

Our patient in this report presented with mixed types of AHS, including a motor type of AHS by the presence of intermanual conflict (callosal type of AHS) and sensory type of AHS by sensory impairment such as alien hand and left hemianesthesia (posterior AHS). The neuroanatomic substrate of AHS and its explanatory models have remained controversial due to the noncircumscribed nature of cerebral injuries present in most cases [12]. Several hypotheses have been suggested, but the most accepted one seems to be the disconnection syndrome [13,14], secondary to the lesions in the medial frontal lobe, the supplementary motor area, anterior cingulate gyrus, medial prefrontal cortex, anterior corpus callosum and posterior parietal lobe. Our patient demonstrated alienation and loss of voluntary control of the left hand, which is consistent with the above anatomical explanation. Our case also provides supportive evidence for damage to the corpus callosum as the anatomic basis of nondominant AHS and conforms to a model of interhemispheric disconnection as the essential component of this unusual behavioral syndrome. We emphasize the importance of distinguishing different features of abnormal behaviors in AHS because it may result in different treatment strategies [15]. However, its significance as a sign of disconnection syndrome is not well validated and needs further investigation.

Neuroimaging studies in our case identified the responsible lesions for the interhemispheric disconnection syndrome in the corpus callosum. This report is explicit in manifestations and the neuroimaging results. The cause of this infarct in our case was possibly due to the atherosclerotic fibrous plaques of the carotid artery, with long-standing hypertension, coronary heart disease and diabetes mellitus of 20 years. As a result, the collective evidences of DWI, MRA should have the advantages of differentiating infarction from other pathologies. Meanwhile, the combination of modern advanced imaging techniques makes the accurate diagnosis of corpus callosal infarction possible, biopsy not necessary any more [16]. In addition, more comprehensively neuropsychological tests are needed to evaluate the brain function. The combinations of functional MRI (such as task-related and resting state fMRI), CT angiography (CTA), CT perfusion (CTP), perfusion weighted imaging (PWI), magnetic resonance spectroscopy (MRS), and digital subtraction angiography (DSA) along with follow-ups should be employed as supplement to the original studies.

## Conclusions

In conclusion, infarcts of the corpus callosum are not common, which is most likely due to its rich blood supply from three main arterial systems, specifically the anterior cerebral, anterior communicating, and posterior cerebral arteries [17]. The isolated infarcts of the anterior cerebral arteries are uncommon (Figure 1f), accounting for only 0.6% of all ischaemic infarcts [18]. As a result, the patient described in our case is unique in exhibiting a complete corpus callosum infarction with a mixed types of AHS, including a motor type of AHS by intermanual conflict (callosal type AHS) and a sensory type of AHS (posterior AHS) by alien hand and left hemianesthesia. To our knowledge, rare reports have ever documented such mixed AHS coexisting secondary to the callosal lesion without evidence of cortical involvement, based on advanced neuroimaging methods as in our case.

## Consent

Written informed consent was obtained from the patient for publication of this case report and accompanying images. A copy of the written consent is available for review by the Editor-in-Chief of this journal.

## Competing interests

The authors declare that they have no competing interests.

## Authors' contributions

JLY examined, evaluated the patient and drafted the manuscript. SKW and XJG performed and interpreted the MRI studies. WLH participated in the design of the case-report and helped to draft the manuscript. All authors read and approved the final manuscript.

## Pre-publication history

The pre-publication history for this paper can be accessed here:

http://www.biomedcentral.com/1471-2377/11/142/prepub
